# Epidemiologic analysis of 8000 acute vertebral fractures: evolution of treatment and complications at 10-year follow-up

**DOI:** 10.1186/s13018-022-03147-9

**Published:** 2022-05-14

**Authors:** Sebastian F. Bigdon, Yannis Saldarriaga, Katharina A. C. Oswald, Martin Müller, Moritz C. Deml, Lorin M. Benneker, Timo M. Ecker, Christoph E. Albers

**Affiliations:** 1 Department of Orthopaedic Surgery and Traumatology, Inselspital, University Hospital, University of Bern, Bern, Switzerland; 2 Department of Emergency Medicine, Inselspital, University Hospital, University of Bern, Bern, Switzerland; 3 Bone and Motion Center , Hirslanden Clinic Lausanne, Lausanne, Switzerland

**Keywords:** Spine fractures, Vertebral fractures, Complications, Revision surgery, Treatment patterns, Spine surgery, Osteoporosis, Epidemiology

## Abstract

**Study design:**

This is a retrospective cohort study.

**Objectives:**

This study aims to determine the proportional incidence, clinical characteristics, treatment patterns with complications and changes in treatment of vertebral fractures over 10 years at a Swiss university hospital.

**Methods:**

A retrospective cohort study was performed. All patients with an acute vertebral fracture were included in this study. The extracted anonymized data from the medical records were manually assessed. Demographic data, exact location, etiology, type of treatment and complications related to the treatment were obtained.

**Results:**

Of 330,225 treated patients, 4772 presented with at least one vertebral fracture. In total 8307 vertebral fractures were identified, leading to a proportional incidence of 25 vertebral fractures in 1000 patients. Fractures were equally distributed between genders. Male patients were significantly younger and more likely to sustain a traumatic fracture, while female patients more commonly presented with osteoporotic fractures. The thoracolumbar junction (Th11-L2) was the most frequent fracture site in all etiologies. More than two-thirds of vertebral fractures were treated surgically (68.6%). Out of 4622 performed surgeries, we found 290 complications (6.3%). The odds for surgical treatment in osteoporotic fractures were two times higher before 2010 compared to 2010 and after (odds ratio: 2.1, 95% CI 1.5–2.9, *p* < 0.001).

**Conclusion:**

Twenty-five out of 1000 patients presented with a vertebral fracture. More than 4000 patients with over 8307 vertebral body fractures were treated in 10 years. Over two-thirds of all fractures were treated surgically with 6.3% complications. There was a substantial decrease in surgeries for osteoporotic fractures after 2009.

## Introduction

Emergency physicians, orthopedic and neurosurgeons, are commonly confronted with patients suffering from spine fractures. Fractures of the spine have a reported incidence of 24–90 cases per 100,000 inhabitants [1, 2]. Despite up-to-date treatment modalities, lengthy rehabilitation, long work absences or permanent invalidity may occur. Consequently, spine fractures are frequently associated with a significant impact on daily living activities, leading to a considerable primary and secondary socioeconomic cost burden [2, 3]. A Swedish study reported health costs related to osteoporotic vertebral fracture of up to 31,545€ per patient in the first year [4]. Furthermore, delayed or missed diagnosis of vertebral fractures may increase morbidity and mortality [5].

The treating physician is additionally facing the challenge of an aging population. For example, one of two women beyond 50 years of age will suffer from an osteoporotic spine fracture [3, 6]. Furthermore, there seems to be a trend for elderly patients suffering from more severe trauma and injuries even without prevalent osteoporosis [7].

Fractures of the spine can occur because of trauma, osteoporosis or a pathological (infection/neoplastic) process [3, 8, 9]. While there is a constant medical evolution in treating tumors, there seems to be no decrease in fracture risk in neoplastic disease of the spine [10].

The treatment of spinal fractures aims to restore or maintain neurologic function and achieve biomechanical stability. There is a high variety of surgical and non-surgical options to accomplish these goals depending on fracture pattern, localization and patient-based criteria.

Knowing the incidence and characteristics of patients with spinal trauma is crucial to determine risk factors, identify trends in treatment and patient-related specifics, and survey the effectiveness and diversity of different treatment modalities.

Concerning osteoporotic fracture therapy, the year 2009 was a breaking point. Since then, there has been a substantial, ongoing controversy about whether patients benefit from minimally invasive surgery (vertebroplasty/kyphoplasty). Two randomized controlled trials published in the New England Journal of Medicine in 2009 showed that patients do not benefit from vertebroplasty [11, 12]. On the other hand, randomized controlled trials published in 2010 revealed the exact contrary, with less pain and faster recovery after vertebroplasty or kyphoplasty [13, 14]. This has been endorsed by an epidemiologic study showing an increased survival after surgery [15]. Since then, a large number of studies have dealt with the subject, but no uniform opinion has prevailed. Considering this discrepancy, it is interesting to discover whether there are changes in treatment with this substantial controversy.

Each etiology of vertebral fracture has its unique epidemiology and characteristic. Up to this point, there is a scarcity in the literature describing and comparing the etiology, characteristics and treatment of a large cohort. This study has the intention to provide a holistic overview of the occurrence of vertebral fractures, their etiology, distribution and treatment with associated complications in a Swiss trauma center.

This study aims to determine (i) the proportional incidence, (ii) clinical characteristics, (iii) treatment patterns with associated complications and (iv) changes in treatment for vertebral fractures over 10 years at a Swiss university hospital.

## Material and methods

### Study design and setting

A retrospective cohort study was performed. All patients were treated at the Department of Emergency Medicine and the Department of Orthopaedic Surgery and Traumatology at the Inselspital, Bern University Hospital, Switzerland. The Inselspital has a catchment area of about two million people; it is the largest level one trauma-center/tertiary referral hospital in the area.

### Eligibility criteria

All patients suffering from an acute vertebral fracture between January 1, 2003, and December 31, 2012, were included in this study. Exclusion criteria were age under 16 years and consultations with incomplete medical records.

### Definitions

Fractures were defined as acute if the onset of symptoms was within three weeks at the point of presentation at our hospital.

The etiology was defined as follows:

- Fractures were defined as *traumatic* if they were caused by an adequate trauma (i.e., fall from height (> 2 m above ground), motor vehicle accident, mountain-related sports).
- *Osteoporotic* fractures were defined as fractures in patients with either pre-known osteoporosis and/or fractures of spontaneous origin, inadequate trauma (fall from standing, lifting or spontaneous fracture without trauma).
- Fractures were defined as *pathological* if they were associated with a spinal tumor or infection.

### Surgical strategy

To decide whether surgery is necessary, a proper fracture classification is essential. Regarding traumatic fractures, we classified all fractures regarding AO Spine Classification [16]. Patients suffering from an A3, A4, B- or C-Type fracture are routinely treated surgically. Regarding osteoporotic fractures, the standard treatment algorithm to determine the necessity for surgery involves fracture pattern (surgery more likely in severe deformity) and pain. Pathological fractures were treated surgically if instability, neurologic deficit or sepsis is present or likely. Fractures of any etiology and type with accompanying neurologic deficit were treated surgically.

### Data extraction and search strategy

All medical records of eligible patients were screened using a defined search algorithm. The algorithm consisted of the keywords “Fraktur,” “Fracture” and “Fx” connected with Boolean operators. Using the clinical information system (Qualicare™, Bern, Switzerland), complete, anonymized data were exported into Microsoft Excel (Microsoft Corporation, USA). After screening the list of diagnoses of all documents matching the algorithm to search for matching patients, a full-text analysis was conducted to extract the information for further analysis.

The abstractor (YS) was trained in regard to inclusion and exclusion criteria before data collection. The abstractor and investigators have not been involved in treating the patients monitored in this study; they were not blinded to the study aims.

The extracted anonymized data from the medical records at admission, after surgery, after discharge and from possible follow-up consultations were manually assessed by one study investigator (YS) and supervised by the corresponding author (SFB) and the senior authors (TE and CEA) for the following items:

(i) Demographical data: gender, age at clinical presentation, year of treatment
(ii) Exact location
(iii) Etiology as defined above
(iv) Type of treatment (surgery and non-operative), if surgery was performed, the exact type of surgery and/or revision (Table 1) and complications (Table 2) related to the treatment.

**Table 1:** Detailed analysis of the surgical techniques performed during the 4129 interventions

Surgical techniques	First intervention	Second intervention	Third intervention	Overall
	Number (%)	Number (%)	Number (%)	Number (%)
Open posterior instrumentation w. decompression/fusion	422 (10.5%)	14 (2.6%)	1 (2.0%)	437 (9.5%)
Open posterior instrumentation w/o decompression with fusion	253 (6.3%)	7 (1.3%)	0 (0%)	260 (5.6%)
Open posterior instrumentation w/o decompression w/o fusion	73 (1.8%)	7 (1.3%)	1 (2.0%)	81 (1.8%)
Open posterior instrumentation w decompression w/o fusion	179 (4.4%)	12 (2.3%)	0 (0%)	191 (4.1%)
Percutaneous posterior instrumentation	90 (2.2%)	3 (0.6%)	0 (0%)	93 (2.0%)
Stand-alone anterior instrumentation	354 (8.8%)	5 (0.9%)	0 (0%)	359 (7.8%)
Combined single-stage anterior−posterior instrumentation	39 (1.0%)	0 (0%)	0 (0%)	39 (0.8%)
Vertebroplasty	2065 (51.1%)	138 (25.9%)	5 (9.8%)	2208 (47.8%)
Balloon kyphoplasty	143 (305%)	9 (1.7%)	0 (0%)	152 (3.3%)
Stentoplasty	138 (3.4%)	7 (1.3%)	0 (0%)	145 (3.1%)
Posterior instrumentation combined with 8, 9 or 10	154 (3.8%)	3 (0.6%)	0 (0%)	157 (3.4%)
Transpedicular vertebral augmentation w. bone graft, bone substitute w/ w/o posterior instrumentation	38 (0.9%)	0 (0%)	0 (0%)	38 (0.8%)
Other (biopsy, nerve root revision, local decompression and Optimesh techniques)	89 (2.2%)	39 (7.3%)	4 (7.8%)	132 (2.9%)
Anterior column reconstruction after posterior instrumentation	1 (0.0%)	203 (38.1%)	1 (2.0%)	205 (4.4%)
Hardware removal	0 (0%)	86 (16.1%)	39 (76.5%)	125 (2.7%)
Total	4038 (100%)	533 (100%)	51 (100%)	4622 (100%)

**Table 2:** Complications of surgical treatment

Complication	Frequency	Percentage (/all complications) (%)	Percentage (/all surgeries) (%)
Implant failure	82	28	1.8
Surgical site infection	58	20	1.3
Neurologic worsening	45	16	1
Delayed skin closure without infection	28	10	0.6
Hematoma	20	7	0.4
Junctional kyphosis	17	6	0.4
Pseudoarthrosis	12	4	0.3
Implant-associated discomfort	12	4	0.3
Leakage of spino-cerebral fluid	6	2	0.1
Implant malpositioning	4	1	0.09
Persistent pain	4	1	0.09
Procedure-based mortality	2	1	0.04
Total	290	100	6.3

To avoid possible bias, a period and cohort in which neither of the personnel involved in this study was involved in the treatment of study participants was reviewed. As no outcome data or variables were obtained, no interrater reliability was performed.

### Statistical methods

Statistical analysis was performed with GraphPad PRISM version 9.1.2 (GraphPad Software, San Diego, California, USA). Normal Gaussian distribution was tested with the Shapiro–Wilk test. For normally distributed data, descriptive statistics are presented as mean ± standard deviation [minimum–maximum]. Non-normally distributed variables are shown as median (interquartile range (IQR) 25% percentile–75% percentile; range minimum–maximum). Logistic regression was used to determine predictive factors for fracture etiology and is shown as (odds ratio (OR); *p* value). Adjusted probability for surgical treatment was calculated using logistic regression and is presented as probability [%] (95% confidence interval lower–upper). Statistical significance was set at *α* ≤ 0.05.

### Ethical considerations

The local ethics committee review board, Bern, Switzerland, approved this study (Ref.-Nr. KEK-BE: 2016-01078), and informed consent was waived.

## Results

**Fig. 1:**
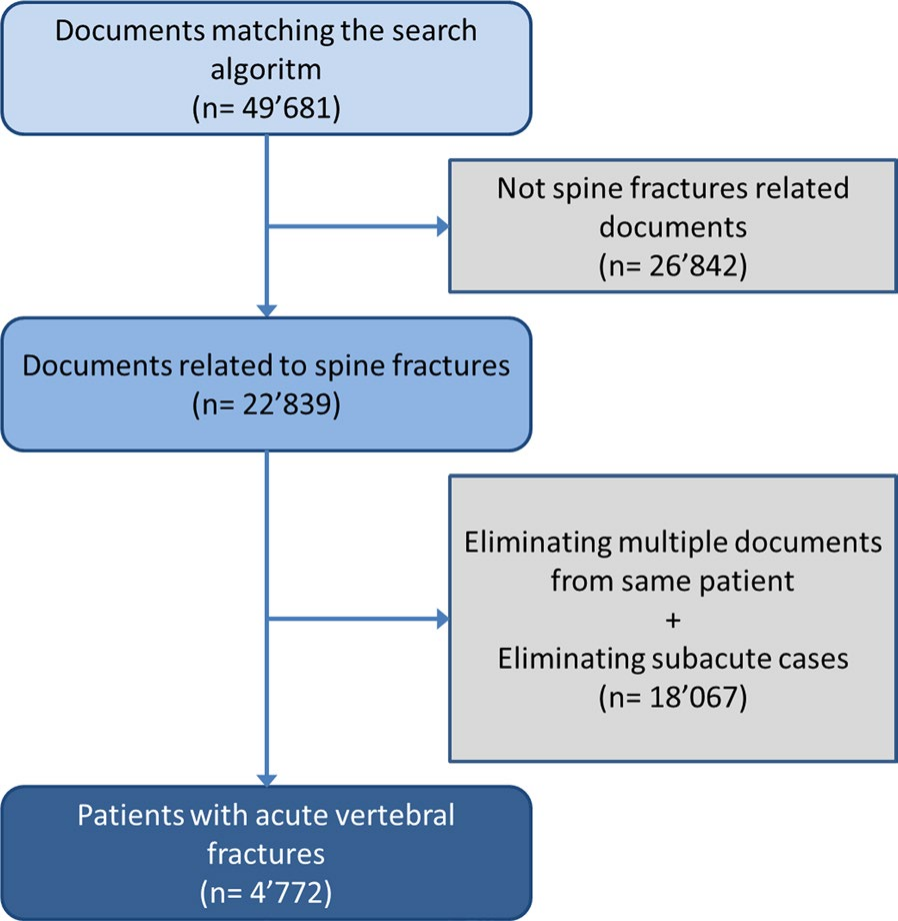
Flowchart: extraction and elimination process

Of 330,225 patients treated at the Emergency and Orthopaedic Department during the study period, 49,681 documents were identified by the search algorithm. Of those, 22,839 were related to spinal fractures. Further exclusion criteria were reports unrelated to acute spinal fractures, multiple documents concerning the same patient, patients age under 16 years and incomplete records. In total, 4772 patients with 8307 acute vertebral fractures were identified (Fig. 1).

### Sociodemographic characteristics

#### Age

The median age was 65 (IQR 47–77, range 16–98) years at the time of the fracture. Regarding traumatic vertebral fractures, the median age was 51 (IQR 35–66, range 16–98) years, 77 (IQR 69–83, range 23–99) years in osteoporotic and 66 (IQR 56–75, range 16–94) years in pathological fractures, respectively.

#### Gender

In total, 49.8% of all patients with vertebral fractures were males, and 50.2% were females. Male patients suffering from a fracture (median age 56 years, IQR 39–70, range 16–98) were significantly younger (*p* < 0.001) than female patients (median age 72 years, IQR 58–80, range 16–97).

### Proportional incidence

Of 330,225 treated patients in the investigated period (302,961 in the emergency department and 27,264 in the spine unit of the department of orthopedic surgery and traumatology), 4772 patients with 8307 vertebral fractures were identified.

Hence, the proportional incidence of vertebral fractures was 25 in 1000 patients presented.

In male patients, a peak of the proportional incidence was observed between 60 and 69 years of age (17.8% of all vertebral fractures in male patients), whereas in female patients, an incidental peak was seen between 70 and 79 years old (29.5% of all vertebral fractures in female patients).

### Clinical characteristics

#### Etiology

Of all spinal fractures, 48.9% of patients had trauma-associated fractures, 40.5% osteoporotic and 10.6% pathological fractures.

**Fig. 2:**
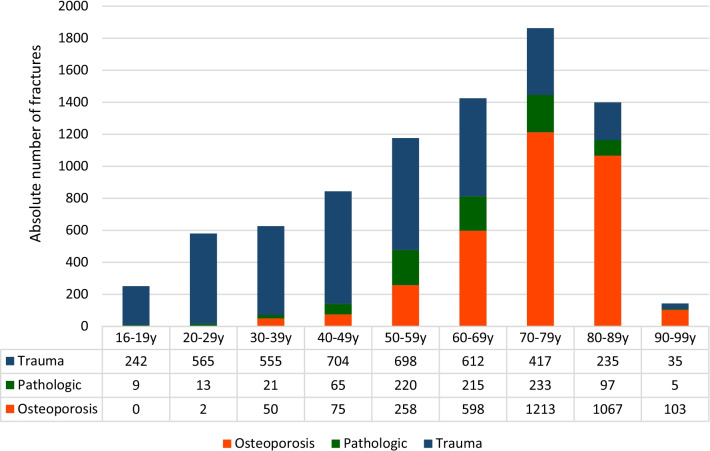
Distribution of age and etiology

The proportional incidence of traumatic fractures decreased with age, while patients treated for osteoporotic fractures were more likely to be over 60 years of age (Fig. 2).

**Fig. 3:**
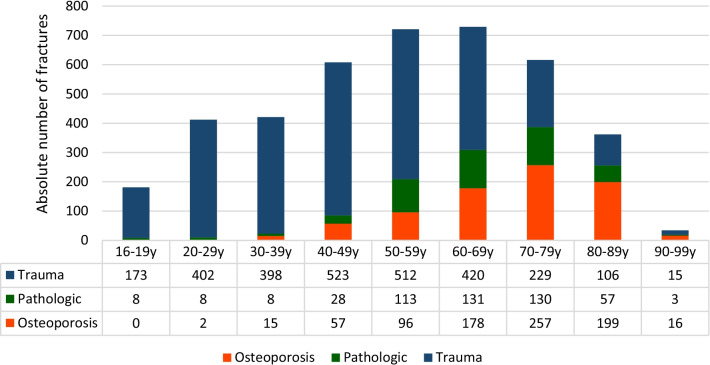
Distribution of age and etiology in male patients

**Fig. 4:**
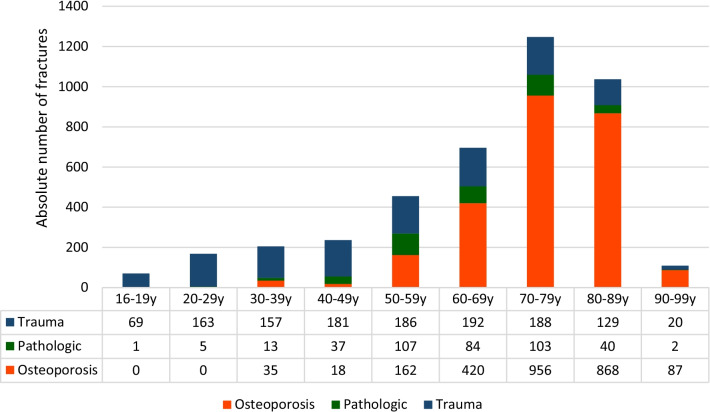
Distribution of age and etiology in female patients

Logistic regression analysis revealed, male patients were more likely to sustain traumatic vertebral fractures (OR 3.89; *p* < 0.0001), whereas females were more likely to suffer from osteoporotic vertebral fractures (OR 5.13; *p* < 0.0001). Male patients had a significantly higher risk of sustaining a pathological fracture (OR 1.28; *p* = 0.006) (Figs. 3, 4).

#### Localization

**Fig. 5:**
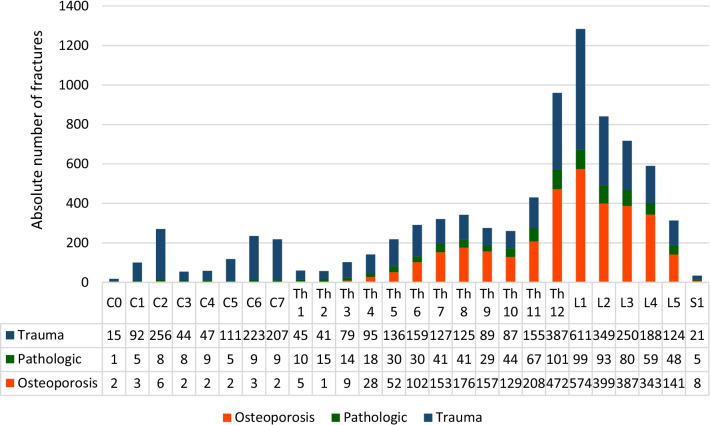
Fracture localization and etiology

The thoracolumbar junction was the most frequent fracture site, with Th11–L2 (32.1%) being most commonly involved in general and in all etiologies separately (Fig. 5).

The cervical spine is prone to traumatic fractures, with C2, C6 and C7 most commonly affected. However, the thoracolumbar region is generally the most prevailing site to suffer from vertebral fractures in general and in all etiologies separately.

**Fig. 6:**
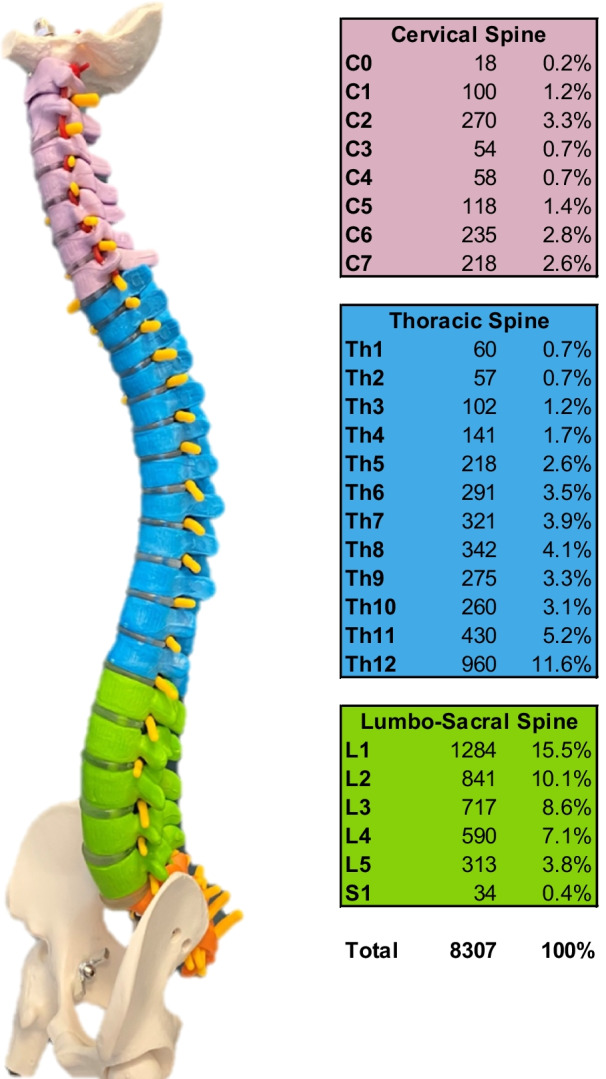
Lateral view of the spinal column with anatomic levels and the detailed distribution of vertebral fractures according to the vertebral level

The cervical spine was affected in 2.1% from osteoporotic fractures, while the distribution of osteoporotic fractures in the thoracic (43.2%) and lumbar (49.0%) spine is comparable to the fracture allocation of traumatic vertebral fractures. Like traumatic and osteoporotic lesions, pathological fractures occurred in 55.2% of the lumbar spine, including the thoracolumbar junction Th12/L1 (Fig. 6).

#### Biomechanical regions

In all etiologies, the thoracolumbar junction (Th11-L2) was the region, where most of the fractures occurred (osteoporosis: 49.1%; pathological: 41.0%; trauma: 40.0%; total: 42.3%) (Fig. 8).

**Fig. 7:**
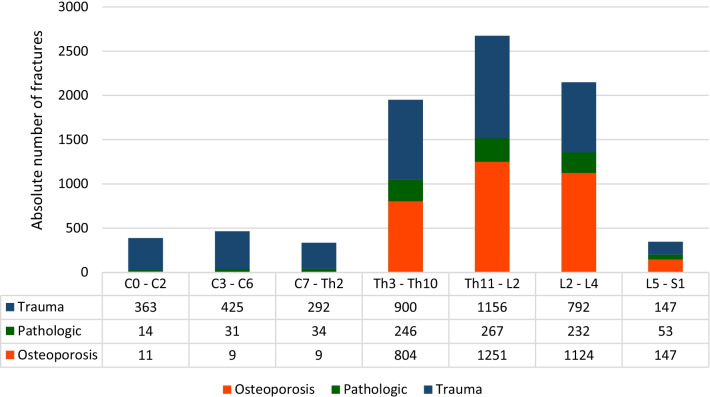
Distribution of fractures according to etiology and biomechanical region

**Fig. 8:**
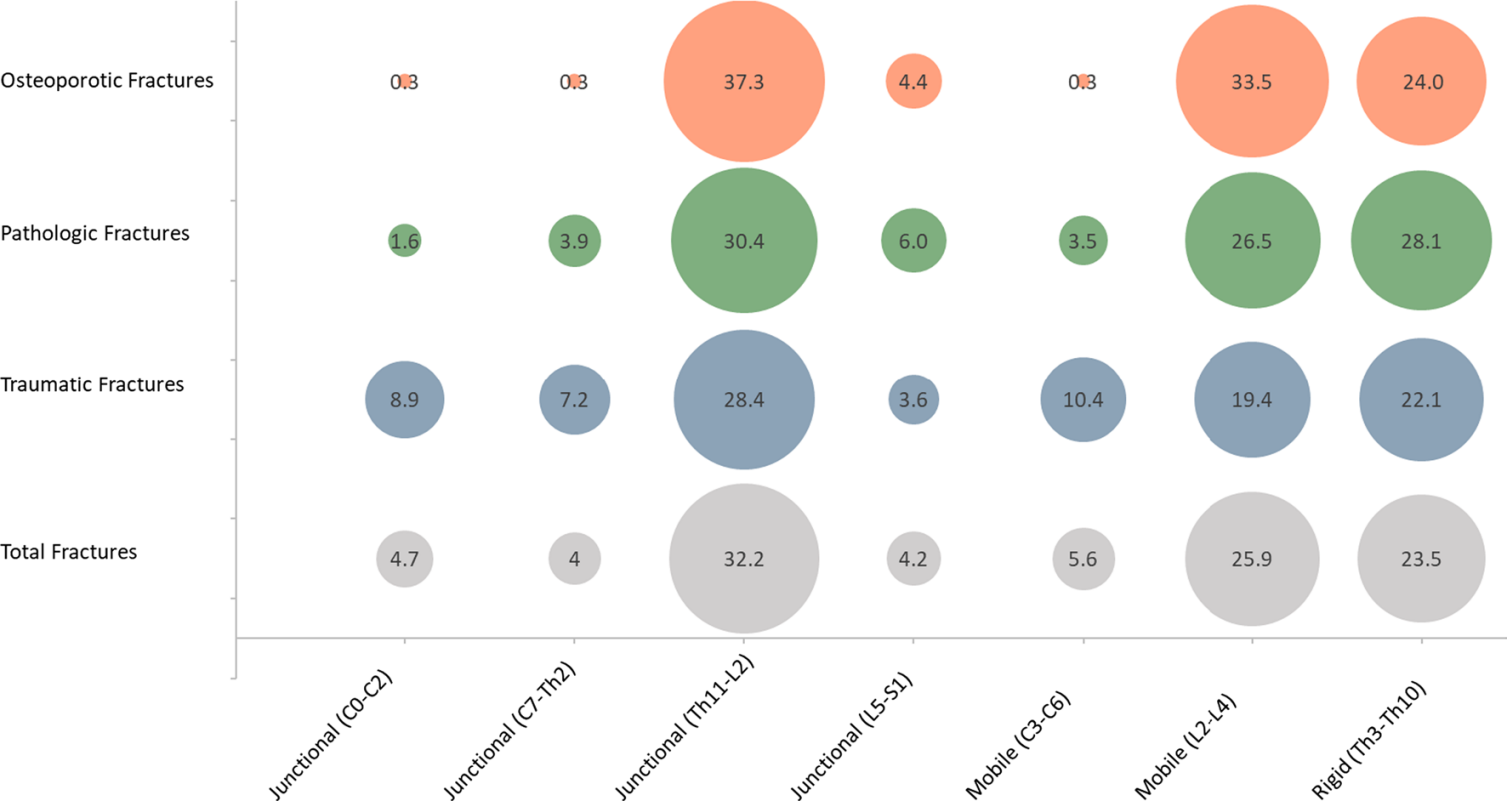
Distribution of fractures according to etiology and biomechanical region (% of total fractures)

Figure 7 shows the distribution of fractures according to fracture region and etiology (Fig. 8).

### Treatment patterns

#### Treatment

**Fig. 9:**
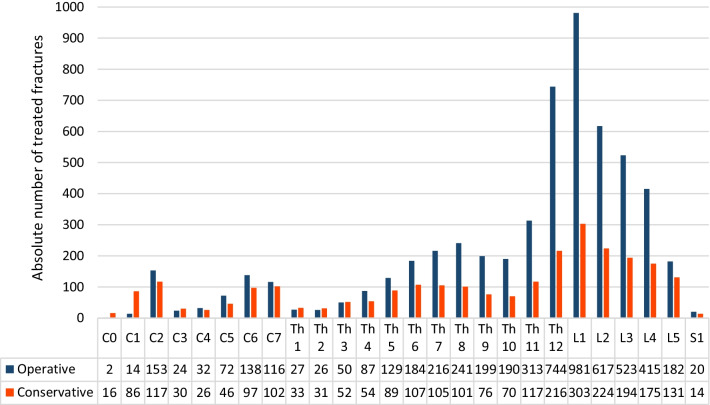
Surgically and conservatively treated fractures according to the fracture level

As mentioned earlier, our surgical strategy is to surgically treat unstable fractures, patients with neurologic impairment and immobilizing pain. This results in more than two-thirds of vertebral fractures being treated surgically (68.6%). Regarding the cervical spine, 51.4% of all fractures were treated surgically and 48.5% conservatively. Fractures in the thoracic spine below Th3 (71.1%), as well as in the lumbar spine (72.4%), were more often treated surgically (Fig. 9).

During the ten-year period, 75.2% of all clinical cases were treated surgically—13.4% of those had a second intervention furthermore, and 1.3% needed a third surgical intervention (revision surgery for complication excluded), adding up to 4129 surgeries. Since multiple surgical techniques are possibly used during one operation, there are more interventions than surgeries (4622 interventions in 4129 surgeries).

In the decade described, the most frequently used intervention was vertebroplasty (47.8%). It was almost exclusively used in osteoporotic and pathological fractures. As expected in traumatic injuries, instrumentations either open or percutaneous with or without decompression or fusion were predominantly used (Table 1).

#### Complications

Out of the 4622 performed surgeries, we found 290 complications revealing an overall complication rate of 6.3% (Table 2). Implant failure was the most frequent complication (28%), followed by surgical site infection (20%) and postoperative neurologic worsening (16%).

### Changes in treatment

Adjusted on age, gender, and multiple-level injuries, the odds for surgical treatment for osteoporotic fractures was two times higher before 2010 compared to 2010 and after (odds ratio: 2.1, 95% CI 1.5–2.9, *p* < 0.001).

**Fig. 10:**
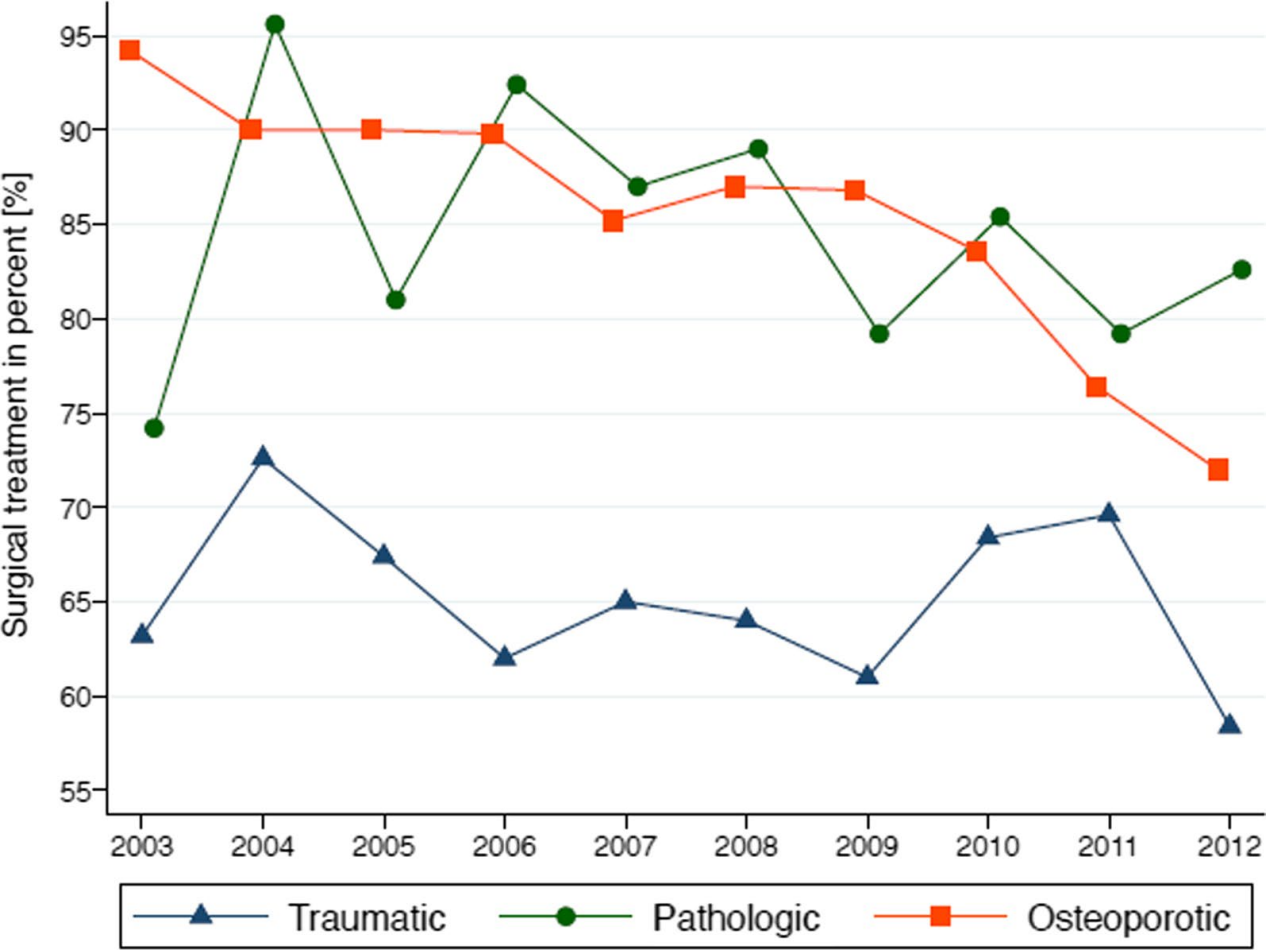
Relative number of operatively treated spine fractures according to spine fracture etiology

From 2003 to 2009, the adjusted probability for surgical treatment of an osteoporotic vertebral fracture was 88.6% (95% CI 87.0–90.2). From 2010 to 2012, the adjusted probability decreased to 79.0% (95% CI 74.1–83.9). This indicates a significant change in treatment strategy after 2009. Figure 10 shows the development of surgically treated spine fractures over the investigated decade.

## Discussion

This study shows the proportional incidence and trends in the management of vertebral fractures in a level one/tertiary referral center during a decade. Up to this point, this is the first study showing a complete overview of patients treated for spine fractures of any etiology over a whole decade at a level one trauma/tertiary referral center for spine surgery. Some studies provide insight and data for the epidemiology of traumatic fractures [17–19] with slightly different findings regarding age and gender distribution. While our cohort sustaining traumatic spine fracture had a mean age of 50 years and a comparably even distribution between gender, the epidemiology presented from the study groups from the USA (mean 43.8 years) [17], China [18] and Ireland (mean 32 years) [19], seems to be younger and males are more commonly affected (m:w ratio: 1.3:1, respectively, 2.33:1). Moreover, they present a comparable frequency for the site of occurrence for traumatic vertebral fractures. Young men seem to be more prone to sustain a high-energy traumatic spine fracture, while fractures associated with minor falls and osteoporosis increase in frequency with age.

Women are at comparably high risk for osteoporotic fractures. Therefore, it seems to be reasonable to look for osteoporosis in female patients over 60 as an underlying disease after a sustained fracture [20]. Patients suffering from a major osteoporotic fracture of the spine have a significantly increased relative risk to sustain another fracture within a short period of time [21].

Regarding osteoporotic fractures, we were able to confirm the study results previously demonstrated. Robinson et al. showed an increased risk for postmenopausal women to sustain an osteoporosis-related vertebral fragility fracture with a similar gender distribution [22]. Oei et al. showed a similar distribution for the location of occurrence as well as gender and age distribution as shown in this study [23].

Literature describing the incidence, fracture sites, as well as gender and age distribution for pathological fractures is scarce and usually limited to a specific cancer [24, 25]. A population-based study from Norway presented by Zaikova et al. showed an annual incidence of 26/100 000 inhabitants for spinal metastatic disease [26] unfortunately without age and gender distribution and further description of the location and whether a fracture was imminent. It remains unclear why male patients suffer more frequently from pathological fractures.

Regarding the fracture site, we found that pathological fractures most commonly occur in the thoracolumbar region. Interestingly this distribution is similar to the distribution of traumatic or osteoporotic fractures. The reason is not apparent. Prior to this study, we anticipated a more even distribution across all vertebral bodies. The reason for this gender and fracture distribution may be an exciting topic to elucidate in further studies.

We performed 4622 surgeries due to traumatic, osteoporotic or pathological fractures in one decade. Since this study was conducted at a tertiary referral center, the more severe fractures and cases are referred to our clinic, granting a higher rate of surgeries.

Reviewing our data, we could show 290 revision surgeries adding up to a total complication rate of 6.3%. This percentage is low compared to previously published data, with complications ranging between 8.4 and 28.8% in smaller cohorts [27–30]. Unfortunately, the definition of complications varies significantly between the different studies.

The most frequent complication we observed was implant failure, followed by surgical site infection and neurologic worsening. Regarding the previously described studies, we had lower numbers for surgical site infection (1.3%) but higher rates for implant failure (1.8%).

When reviewing the data carefully, the most exciting trend seems to be a decrease in surgical intervention in osteoporotic fractures after 2009. This may be because of a strong and ongoing discussion in the literature and spine societies starting around 2009. While two randomized controlled trials (RCT) in 2009 showed no benefit of vertebroplasty [11, 12], three other RCTs [13, 14, 31] showed significantly less pain and decreased mortality after vertebroplasty. This discussion seemed to influence the treatment of osteoporotic spine fractures. The adjusted probability for surgery decreased by nearly 10% after 2009.

Meanwhile, there are numerous studies and randomized controlled trials regarding the treatment of osteoporotic spine fractures. Due to the ongoing dispute, there is a strong need for better comparability and validated treatment algorithms. A first step was made by proposing a new fracture classification by Schnake et al. [32] in 2018. The challenge for the future remains to determine the patients in which surgery is beneficial over patients in which a conservative approach is equally good.

### Limitations

With this study, we describe the largest single-center cohort over a decade. We avoid observer and selection bias because no surgeon, who has been involved in the treatment, is still working at our hospital.

However, since we can only report from a single center, we can only report the proportional incidence regarding the patients treated at our tertiary referral hospital. There is no registry for spinal injuries yet to detect the exact incidence. Moreover, because of the changes with our clinical information system we are only able to provide ten-year data up to 2012.

Patients referred to our center are usually more severely injured; hence, surgery is more often needed. This fact may lead to selection bias. The data were collected retrospectively. The conclusiveness of retrospective data is limited.

Regarding the etiology of fractures, there is a possibility for misclassification bias. Since osteoporosis is endemic in Switzerland, some osteoporotic fractures may be misclassified as traumatic if the inclusion criteria as cited above happened to apply. We do not collect DXA values as a standard operational procedure in patients.

Since the exact classification was documented inconsistently and insufficiently over the ten year study period and not all radiographic data are still available, we are not able to provide the precise allocation of fracture severity according to our usual classification system (AO Spine Classification)^16^.

## Conclusion

Twenty-five out of 1000 patients presented with a vertebral fracture in our Swiss-level one trauma-center/tertiary referral hospital in Bern. More than 4000 patients with over 8307 vertebral body fractures were treated at a Swiss university hospital in 10 years. The distribution of fractures was equal between genders, with male patients being significantly younger than female patients at the time of the incident. More than two-thirds of all fractures were treated with surgery, with 6.3% complications. There was a substantial decrease in surgical interventions in osteoporotic spine fractures after 2009.

## Data Availability

Please contact the author for data requests.

## References

[1] den Ouden LP, Smits AJ, Stadhouder A, Feller R, Deunk J, Bloemers FW (2019). Epidemiology of spinal fractures in a level one trauma center in the Netherlands: a 10 years review. Spine.

[2] Tian Y, Zhu Y, Yin B (2016). Age- and gender-specific clinical characteristics of acute adult spine fractures in China. Int Orthop.

[3] Kim BG, Dan JM, Shin DE (2015). Treatment of thoracolumbar fracture. Asian Spine J.

[4] Borgström F, Zethraeus N, Johnell O (2006). Costs and quality of life associated with osteoporosis-related fractures in Sweden. Osteoporos Int.

[5] Wang H, Liu X, Zhao Y (2016). Incidence and pattern of traumatic spinal fractures and associated spinal cord injury resulting from motor vehicle collisions in China over 11 years: an observational study. Medicine (Baltimore).

[6] Lippuner K, Johansson H, Kanis JA, Rizzoli R (2009). Remaining lifetime and absolute 10-year probabilities of osteoporotic fracture in Swiss men and women. Osteoporos Int J Establ Result Coop Eur Found Osteoporos Natl Osteoporos Found USA.

[7] Go KT, Cheng JY, Seah X, Goh MH, Teo LT, Cole E (2019). The changing epidemiology of serious trauma in the elderly population: an increasing concern of a tertiary hospital in Singapore. Ann Acad Med Singapore.

[8] Jacobsen SJ, Cooper C, Gottlieb MS, Goldberg J, Yahnke DP, Melton LJ (1992). Hospitalization with vertebral fracture among the aged: a national population-based study, 1986–1989. Epidemiol Camb Mass.

[9] Cooper C, Atkinson EJ, O’Fallon WM, Melton LJ (1992). Incidence of clinically diagnosed vertebral fractures: a population-based study in Rochester, Minnesota, 1985–1989. J Bone Miner Res Off J Am Soc Bone Miner Res.

[10] Oortgiesen BE, Driessen JHM, Hoogendoorn M (2020). No decrease in fracture risk despite 15 years of treatment evolution for multiple myeloma patients: a Danish nationwide case-control study. Bone.

[11] Buchbinder R, Osborne RH, Ebeling PR (2009). A randomized trial of vertebroplasty for painful osteoporotic vertebral fractures. N Engl J Med.

[12] Kallmes DF, Comstock BA, Heagerty PJ (2009). A randomized trial of vertebroplasty for osteoporotic spinal fractures. N Engl J Med.

[13] Klazen CAH, Lohle PNM, de Vries J (2010). Vertebroplasty versus conservative treatment in acute osteoporotic vertebral compression fractures (Vertos II): an open-label randomized trial. Lancet Lond Engl.

[14] Wardlaw D, Cummings SR, Van Meirhaeghe J (2009). Efficacy and safety of balloon kyphoplasty compared with non-surgical care for vertebral compression fracture (FREE): a randomized controlled trial. Lancet Lond Engl.

[15] Edidin AA, Ong KL, Lau E, Kurtz SM (2011). Mortality risk for operated and nonoperated vertebral fracture patients in the medicare population. J Bone Miner Res Off J Am Soc Bone Miner Res.

[16] Magerl F, Aebi M, Gertzbein SD, Harms J, Nazarian S (1994). A comprehensive classification of thoracic and lumbar injuries. Eur Spine J Off Publ Eur Spine Soc Eur Spinal Deform Soc Eur Sect Cerv Spine Res Soc.

[17] Leucht P, Fischer K, Muhr G, Mueller EJ (2009). Epidemiology of traumatic spine fractures. Injury.

[18] Liu P, Yao Y, Liu M (2012). Spinal trauma in mainland China from 2001 to 2007: an epidemiological study based on a nationwide database. Spine.

[19] Lenehan B, Boran S, Street J, Higgins T, McCormack D, Poynton AR (2009). Demographics of acute admissions to a National Spinal Injuries Unit. Eur Spine J Off Publ Eur Spine Soc Eur Spinal Deform Soc Eur Sect Cerv Spine Res Soc.

[20] Ferrari S, Lippuner K, Lamy O, Meier C (2020). 2020 recommendations for osteoporosis treatment according to fracture risk from the Swiss Association against Osteoporosis (SVGO). Swiss Med Wkly.

[21] Kanis JA, Johansson H, Odén A (2018). Characteristics of recurrent fractures. Osteoporos Int J Establ Result Coop Eur Found Osteoporos Natl Osteoporos Found USA.

[22] Robinson WA, Carlson BC, Poppendeck H (2020). Osteoporosis-related vertebral fragility fractures: a review and analysis of the American Orthopaedic Association’s own the bone database. Spine.

[23] Oei L, Koromani F, Breda SJ (2018). Osteoporotic vertebral fracture prevalence varies widely between qualitative and quantitative radiological assessment methods: the Rotterdam study. J Bone Miner Res Off J Am Soc Bone Miner Res.

[24] Nieder C, Haukland E, Pawinski A, Dalhaug A (2010). Pathologic fracture and metastatic spinal cord compression in patients with prostate cancer and bone metastases. BMC Urol.

[25] Silva GT, Bergmann A, Thuler LCS (2015). Incidence, associated factors, and survival in metastatic spinal cord compression secondary to lung cancer. Spine J Off J North Am Spine Soc.

[26] Zaikova O, Giercksky KE, Fosså SD, Kvaløy S, Johannesen TB, Skjeldal S (2009). A population-based study of spinal metastatic disease in South-East Norway. Clin Oncol R Coll Radiol G B.

[27] Reis RC, de Oliveira MF, Rotta JM, Botelho RV (2015). Risk of complications in spine surgery: a prospective study. Open Orthop J.

[28] Camino Willhuber G, Elizondo C, Slullitel P (2019). Analysis of postoperative complications in spinal surgery, hospital length of stay, and unplanned readmission: application of Dindo-Clavien classification to spine surgery. Glob Spine J.

[29] Nasser R, Yadla S, Maltenfort MG (2010). Complications in spine surgery. J Neurosurg Spine.

[30] Schwab FJ, Hawkinson N, Lafage V (2012). Risk factors for major peri-operative complications in adult spinal deformity surgery: a multi-center review of 953 consecutive patients. Eur Spine J Off Publ Eur Spine Soc Eur Spinal Deform Soc Eur Sect Cerv Spine Res Soc.

[31] Van Meirhaeghe J, Bastian L, Boonen S, Ranstam J, Tillman JB, Wardlaw D (2013). A randomized trial of balloon kyphoplasty and non-surgical management for treating acute vertebral compression fractures: vertebral body kyphosis correction and surgical parameters. Spine.

[32] Schnake KJ, Blattert TR, Hahn P (2018). Classification of osteoporotic thoracolumbar spine fractures: recommendations of the spine section of the German Society for Orthopaedics and Trauma (DGOU). Glob Spine J.

